# Chemoprevention of metastasis

**DOI:** 10.18632/oncotarget.2382

**Published:** 2014-08-24

**Authors:** Nancy J. Emenaker, Enrique Zudaire, Brad St. Croix

**Affiliations:** Tumor Angiogenesis Section, Mouse Cancer Genetics Program (MCGP), National Cancer Institute (NCI) at Frederick, NIH, Frederick, MD, USA

Cancer chemoprevention employs natural, synthetic, or biologic substances to reverse, suppress, or prevent the development of cancer. Initially these agents were developed to reduce cancer risk or retard its development, but were not intended to treat cancer. Celecoxib, a selective cyclooxygenase-2 (COX-2) inhibitor, is used clinically to prevent polyp formation in familial adenomatous polyposis (FAP) patients, a population at high risk for colorectal cancer development. However, recent preclinical studies suggest that chemopreventive agents like celecoxib may have effective anti-tumor and anti-metastatic properties against advanced stage disease. Currently, a randomized clinical trial is underway to determine if celecoxib can indeed enhance the therapeutic value of oxaliplatin, leucovorin and fluorouracil in the adjuvant setting of resected colorectal cancer (CALGB 80702; NCT01150045) [1].

**Figure 1:**
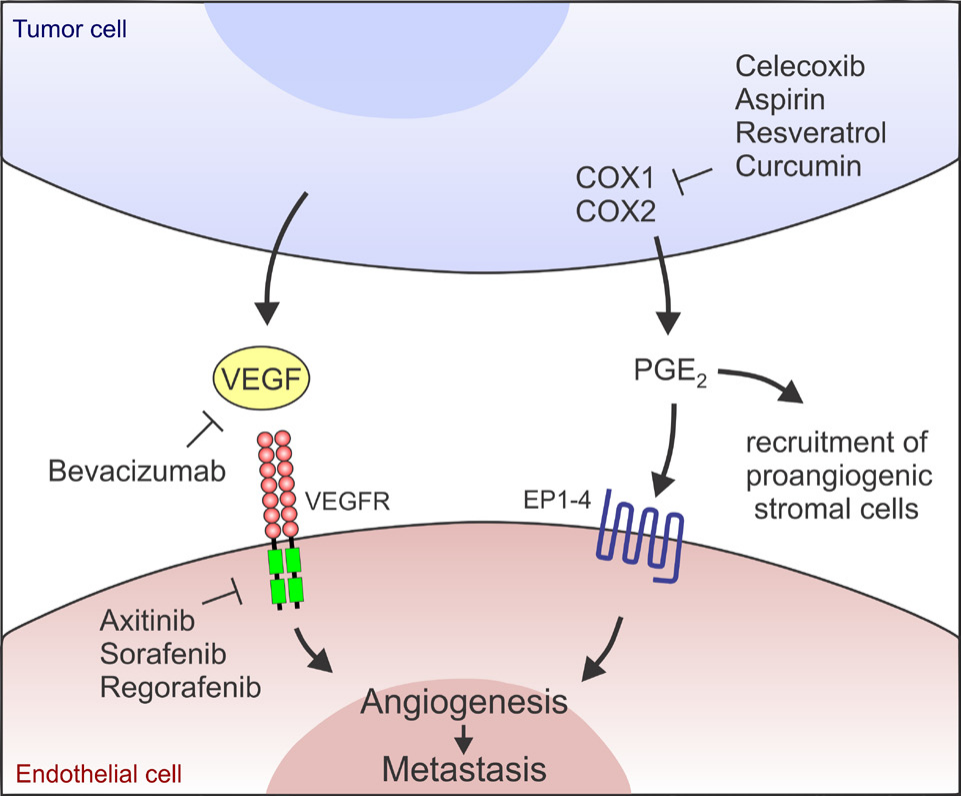
A model highlighting the independent nature of the VEGF/VEGFR and COX/PGE_2_ pathways Dual inhibition of the pathways at the indicated junctures may result in the most effective inhibition of angiogenesis and metastasis.

Recent preclinical tumor studies suggest that the effectiveness of celecoxib against late stage colon and breast cancers can be significantly enhanced when combined with an anti-angiogenic agent (i.e., bevacizumab, neutralizing anti-VEGF antibody) or a receptor tyrosine kinase inhibitor (i.e., axitinib, selectively targeting VEGF receptors) [2] (Figure 1). COX-2 played a key regulatory role in prostaglandin E_2_ (PGE_2_) biosynthesis, driving angiogenesis in CT26 VEGF inhibitor refractory colon tumors. Moreover, activation of the COX-2/PGE_2_ pathway also enhanced myeloid cell recruitment into tumors likely increasing production of additional pro-angiogenic factors and further fueling angiogenesis. PGE_2_ has also been found to promote tumor cell growth, and prevent apoptosis and immune suppression. Thus, PGE_2_ likely promotes tumor growth through multiple mechanisms.

Because tumor vessels provide an escape route for tumor cells, we hypothesized that simultaneous targeting of the VEGF and COX-2/PGE_2_ pathways may also aid in blocking metastases. Indeed, in both experimental and spontaneous colon and breast cancer models dual VEGF/ COX-2 pathway inhibition reduced metastasis [2]. For example, six months post axitinib/celecoxib therapy 60% of the mice were cured by the combination compared to only 8% (axitinib) or 17% (celecoxib) in the monotherapy arms. These studies highlight the potential advantage of combinatorial approaches using anti-angiogenic agents with the chemopreventive agent celecoxib for treating metastases.

Celecoxib is the only clinically approved chemoprevention agent used for high risk FAP patients. While potential cardiotoxicities prevent its chemopreventive use in the general population, as an adjuvant therapy for blocking recurrence of late stage disease the potential benefits may be worth the risk, at least for some patients. Indeed, re-analysis of six large randomized placebo-controlled clinical trials and 72 epidemiological studies found no evidence of celecoxib induced cardiotoxicity in patients with a low baseline cardiovascular risk [3].

As with other targeted agents, identifying patients most likely to respond to VEGF and/or COX-2 inhibition will be a key factor influencing the success of future clinical trials in this area. Appropriate patient stratification would help ensure maximal responses and prevent potential toxicities in individuals unlikely to respond. Unfortunately, in the angiogenesis field reliable, predictive bioassays for directly measuring the responsiveness of different tumor types to anti-angiogenic agents are not yet available. While it is also difficult to predict who will respond to COX-2 inhibition, some studies suggest greater benefit is obtained when tumors overexpress COX-2. Mutations in *PIK3CA* activate the phosphatidylinositol 3-kinase (PI3K) pathway and are present in approximately 15 to 20% of colorectal cancers, resulting in higher COX-2 expression and predicting colorectal cancer responsiveness to aspirin, a non-selective COX-1 and COX-2 inhibitor [4]. Mutations in the gene encoding 15-hydroxyprostaglandin dehydrogenase (15-PGDH), the enzyme responsible for PGE_2_ degradation, can also lead to high local PGE_2_ levels making colorectal cancer resistant to celecoxib therapy [5]. Thus, tumors which harbor activating *PIK3CA* mutations and maintain wildtype *15-PGDH* may be the most likely to benefit from celecoxib or other non-steroidal anti-inflammatory drugs (NSAIDs).

What are the future possibilities for COX-2 inhibition in individuals with an elevated risk for cardiovascular events? One possibility may be to use other less cardiotoxic NSAIDs like aspirin or naproxen. However, these NSAIDs also inhibit COX-1 imputing increased risks for gastrointestinal and renal toxicities. Another possibility may be to substitute COX inhibitors for other tumoricidal agents such as resveratrol, curcumin, vitamin D, naringenin or their synthetic derivatives, which are well tolerated and have been shown to block COX-2 expression. Although soluble epoxide hydrolase (sEH) inhibitors used as a monotherapy may promote angiogenesis, when combined with celecoxib they also appear to potently suppress angiogenesis, tumor growth, and metastasis [6]. Similarly, COX-2/sEH dual pharmacological inhibitors have shown promising antitumor activity in preclinical studies [6]. Importantly, combination COX-2/sEH therapy may circumvent the cardiotoxicities associated with COX-2 inhibition by maintaining cardioprotective prostacyclin (PGI_2_) to thromboxane A_2_ (TXA_2_) ratios. [6]. Therefore, clinically approved agents such as sorafenib and regorafenib, which inhibit VEGFRs and are also among the most potent sEH inhibitors ever identified [7], are promising agents to test in combination with celecoxib. Such combinatorial therapies could potentially help in the prevention or treatment of metastasis while minimizing cardiotoxicities.
